# Interlayer Simplified Depth Coding for Quality Scalability on 3D High Efficiency Video Coding

**DOI:** 10.1155/2014/841608

**Published:** 2014-03-16

**Authors:** Mengmeng Zhang, Hongyun Lu, Huihui Bai

**Affiliations:** ^1^North China University of Technology, No. 5 Jin Yuanzhaung Road, Shijingshan District, Beijing 100144, China; ^2^Beijing Jiaotong University, No. 3 Shangyuancun, Haidian District, Beijng 100044, China

## Abstract

A quality scalable extension design is proposed for the upcoming 3D video on the emerging standard for High Efficiency Video Coding (HEVC). A novel interlayer simplified depth coding (SDC) prediction tool is added to reduce the amount of bits for depth maps representation by exploiting the correlation between coding layers. To further improve the coding performance, the coded prediction quadtree and texture data from corresponding SDC-coded blocks in the base layer can be used in interlayer simplified depth coding. In the proposed design, the multiloop decoder solution is also extended into the proposed scalable scenario for texture views and depth maps, and will be achieved by the interlayer texture prediction method. The experimental results indicate that the average Bjøntegaard Delta bitrate decrease of 54.4% can be gained in interlayer simplified depth coding prediction tool on multiloop decoder solution compared with simulcast. Consequently, significant rate savings confirm that the proposed method achieves better performance.

## 1. Introduction

In January 2013, the Joint Collaborative Team on Video Coding has finalized a final draft about the next generation video standard, that is, High Efficiency Video Coding (HEVC) [1]. Scalable High Efficiency Video Coding (SHVC) and 3D video coding are being formulated as the extensions of HEVC [2]. Given the special performance of HEVC in delivering the target resolution and frame rates [3], 3D video coding and SHVC are also applied to a variety of consumer domains with their different application opportunities. Currently, 3D video coding and scalable video coding are entering broad and possibly sustainable mass markets. Magnificent and excellent three-dimensional scenes in 3D TV can be provided with the mature 3D video coding technology. Scalable video coding is applied to cope with the heterogeneity of networks and devices used in the video service environment. With the rapid evolution in theory and techniques, the technology of scalable video coding based on the 3D video coding may be applied to 3D TV or mobile terminal in the future. Therefore, the investigation of a scalable 3D video coding scenario is important and necessary.

Generally, scalable video coding is a highly attractive solution to the problems caused by the characteristics of modern video transmission systems. The scalable video coding method can be used to achieve the adaptation of a bitrate with features such as temporal and spatial scalabilities [4]. The quality scalability could be treated as a special case of spatial scalability with the same resolution in different layers [5]. This paper proposes a quality scalable 3D video coding that is equipped with both quality scalability and 3D visualization and modified based on the 3D video coding to reduce complexity. Different layers of several simultaneous views are coded into the bitstream for quality scalable 3D video coding.

Depth-Image-Based Rendering (DIBR) is widely used for view synthesis in 3D video coding. Thus, the texture video and the associated depth map are required to be scaled simultaneously [6]. DIBR notes that the execution of proposed scalable methods can be approached in terms of two considerations in this paper. First, an interlayer texture Prediction mechanism will be employed to eliminate the redundancy of the different layers on multiloop decoding solution that was proposed in Van Wallendael et al. [7]. The inter-layer texture prediction mechanism will be utilized in both texture videos and depth maps. This method achieves higher compression efficiency, yet it maintains backwards compatibility with multiple views coded by HEVC. Second, an extraordinary interlayer prediction tool called an interlayer simplified depth coding (interlayer SDC) is used to reduce interlayer redundancy. As depth maps exhibit unique characteristics such as piecewise smooth regions bounded by sharp edges at depth discontinuities [8], new coding tools are required to approximate these signal characteristics [9]. These coding tools include simplified depth coding (SDC) and depth modeling modes (DMM) [10]. Moreover, all the results are tested in an all intraconfigurations as every frame includes three views (view 0 is predicted in I slice, and view 1 and view 2 are predicted in P slice), and the SDC is only chosen in intraframe.

The rest of this paper is organized as follows.  Section 2 introduces the 3D high efficiency video coding. The details of the proposed interlayer SDC tool are presented in Section 3.  Section 4 describes the test scenarios and presents the analysis results.  Section 5 concludes the paper.

## 2. 3D High Efficiency Video Coding

**Figure 1:**
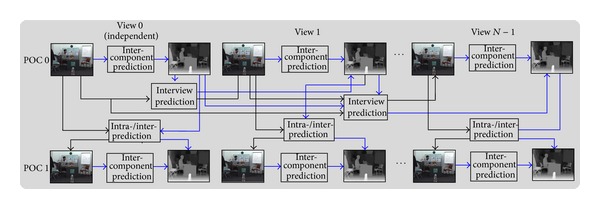
Basic encoder structure with interview and intercomponent prediction.

As one of the extensions of HEVC, the upcoming 3D video coding makes use of the efficient single-view coding tools used in HEVC. HEVC is the latest video coding standard developed by a joint effort between ISO/IEC and ITU-T and succeeding H.264/AVC. This design still follows a traditional hybrid coding approach [9], such as interprediction based on the motion compensated, interprediction residuals of the two-dimensional transform, and quadtree. 3D HEVC is achieved by coding each video view and associated depth map component using a 2D video coding structure that is based on the technology of HEVC. In order to provide backward compatibility with 2D video services, the independent view is coded using a fully HEVC compliant codec [11]. Except that intra-/interframe prediction is still exiting in 3D HEVC, interview prediction for views and intercomponent prediction for views and maps are added into the 3D extension. The prediction structure is depicted in Figure 1 in detail. The blue arrows denote the prediction for depth maps, and the black shows the prediction between the views.

## 3. Proposed Quality Scalability on 3D Video Coding

### 3.1. The Framework of Scalability on 3D Video Coding

**Figure 2:**
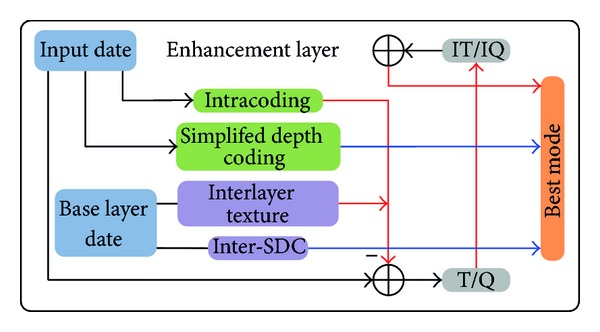
High-level block diagram of the encoder.

The proposed quality scalable scheme employs interlayer simplified depth coding and interlayer texture prediction to remove interlayer redundancy based on the multiloop decoder structure. The multiloop decoding solution is integrated into the proposed scenario as a whole framework of the scalable 3D video coding. The interlayer texture prediction is considered as a basic prediction mode that the base layer (BL) needs to be decoded entirely before the enhancement layers are reconstructed. The transform and quantization processes of interlayer texture prediction predict that CUs are the same as an intrapredicted CU on the QP of the enhancement layer, in which discrete sine transform and discrete cosine transform are applied to the different types of TUs.  Figure 2 depicts the block diagram of the proposed interlayer prediction in encoder. The color-marked parts represent the prediction signalling mechanisms for depth maps in the same view in enhancement layer. The red-marked arrows show the prediction process with transform and quantization, and the blue-marked arrows denote the prediction process without transform and quantization. All modes, including traditional prediction modes and interlayer prediction modes, could be chosen as the best prediction mode by rate distortion optimization (RDO) [12] for texture views and virtual synthesis optimization (VSO) [13] for depth maps. Details of the proposed scalable algorithms are described in the following.

### 3.2. Feasibility Analysis of Interlayer Simplified Depth Coding

**Figure 3:**
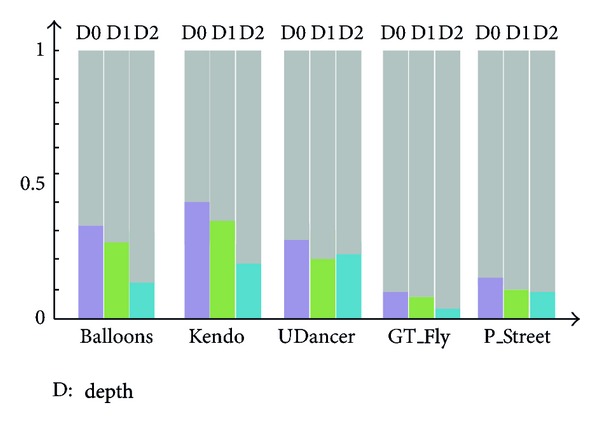
Percentages of SDC-coded block in 300 I-frames.

As an alternative intracoding mode, the SDC approach is added into the intracoded block, and the prediction mode is still INTRA for an SDC-coded block with additional SDC_Flag signals in depth map coding [14]. The information of base layer SDC-blocks can be directly used as the reference for enhancement layer because the clear textural feature leads to many distortionless SDC-blocks, and the dynamic quantization parameter (QP) does not affect the distortion of SDC-blocks as a result of no transform and quantization. The amount of available SDC-blocks becomes an issue of crucial importance as only the distortionless SDC-blocks could be the reused for interlayer SDC. Thus, more attention is paid to the amount of SDC-blocks that could be applied to interlayer SDC. A statistical experiment was then performed. The percentages of distortionless SDC-blocks in all the blocks were calculated in 300 I-frames (Figure 3). Figure 3 shows that the number of distortionless SDC-blocks occupies a large proportion in all depth blocks in the experiment.

### 3.3. Interlayer Simplified Depth Coding Prediction Tool

**Figure 4:**
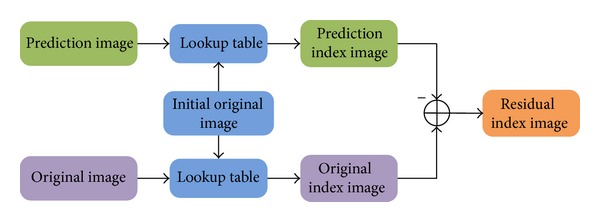
The Structure of the simplified depth coding algorithm.

The interlayer SDC tool is sufficient in generating a good prediction signal and eliminates ringing artifacts for SDC-coded blocks. Instead of coding quantized transform coefficients, the following three parts of information are coded in SDC-coded blocks. The first part is the type of segmentation/prediction of the current block with possible values of DC, DMM, or Planar. The second part is the additional prediction information when the DMM mode is selected as the type of segmentation. The third part is the residual value for each resulting segment, which is present in the original, uncompressed depth map using a depth lookup table (DLT). Initial analysis shows that the DLT is constructed by analyzing a certain number of frames of the input sequence before coding. The residual is obtained from the difference of the prediction index and the original index according to the DLT. The structure of the algorithm is described as follows (Figure 4).

The interlayer SDC utilizes the decompressed date from the collocated distortionless SDC-coded CUs due to the characteristics of distortionless SDC-coded CUs in the base layer. This paper considers the interlayer SDC method as an extension of the intracoding mode, in which the type of segmentation/prediction, additional prediction information, and residual value of the index are obtained from the corresponding base layer CU. The prediction image of the current enhancement layer is rebuilt based on the type of segmentation/prediction and additional prediction information, which have been selected by the traditional SDC in the base layer. The reconstructed image is derived from the residual value of the index and the index prediction image. No transform and quantization processes occur in the interlayer simplified depth coding prediction method, in which only the information of the SDC in the base layer needs to be decoded for the enhancement layer. Thus, no additional data are transmitted for the enhancement layer except a flag of the interlayer SDC mode in the proposed interlayer SDC tools. When the “Inter_SDC_Flag” that is signaled in the enhancement layer is set to be true, the current block is decoded in an interlayer SDC decoder. The “SDC_Flag” is the coding identifier of the traditional SDC for 3D video coding. The “SDC_Flag” and “Inter_SDC_Flag” are used to codetermine the decoding mode in the enhancement layer.  Table 1 shows the detailed implications of identifiers.

Moreover, the DLT in the base layer is used in the inter-layer SDC. The RD cost of interlayer texture prediction and the interlayer SDC method is calculated for the enhancement layer CUs in addition to the RD selection procedure used in unmodified 3D video encoder. The optimal prediction mode is selected to minimize the cost function:

(1) $$\begin{array}{c} {\text{Cost}}_{\text{mode}}=\min\left\{\begin{array}{c} \text{RD}\left(\text{Inter-layer}\;\text{texture}\;\text{prediction}\right)\\ \text{RD}\left(\text{Intra},\text{Traditional}\;\text{SDC}\right)\\ \text{RD}\left(\text{Inter-layer}\;\text{SDC}\right) \end{array}\right\}, \end{array}$$

where Cost_mode_ is the minimalized rate-distortion cost of the current mode. The RD (·) is the rate-distortion cost of every mode by the RDO (VSO).

## 4. Results and Analysis

The scalable 3D video coding theme is implemented based on HTM 6.0 [15]. The common test configurations are defined in D1100 [16]. Two layers (one base layer and one enhancement layer) and three views are simultaneously evaluated in all I-frames. The base layer and enhancement layer are encoded in different QPs with a spatial ratio of 1 : 1. The quantization parameters of base and enhancement texture views are Q1 and Q2. The common conditions specify the biggish Q1 (30, 35, 40, and 45), and the delta QP between two layers is 5. The QPs of depth maps change according to the QP of the associated texture view in 3D video coding. Two experimental methods of displays intuitively describe the experimental results, namely, the PSNR-Bitrate graph and the BD rate table. Comparing the results, the bitrate contains all the layers, and the PSNR is the highest enhancement layer with Q2. Moreover, all the experimental results come from the synthetic view after scaling the texture views and depth maps. Three schemes were realized in this experiment for comparison, namely, single-loop, simulcast coding, and inter-SDC prediction in multiloop solution. The simulcast solution that two layers were both coded in 3D video without the interlayer prediction will be used as the anchor to evaluate our proposed scalable scenario.

**Figure 5:**
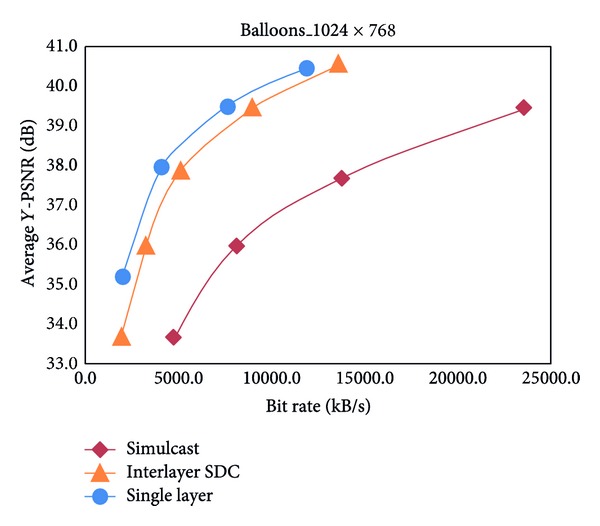
Performance comparison for balloons.

**Figure 6:**
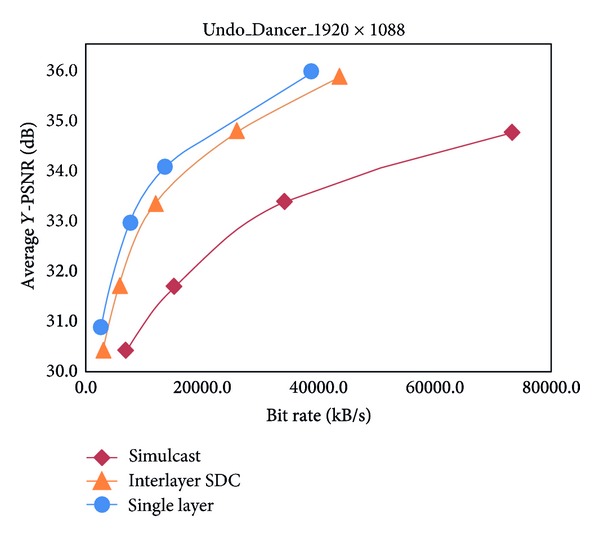
Performance comparison for Undo_Dancer.

The simulcast and the scalable scenarios in interlayer SDC prediction with interlayer texture prediction are compared in Table 2. The results indicate a 55.2% Y-BD-rate decrease for 2 synthesized views, whereas a 54.4% Y-BD-rate decrease is noted for 3 synthesized views. Thus, the overall experimental results of Table 2 show that the proposed interlayer SDC is an inevitable tool for improving compressive performance. Figures 5 and 6 show the efficiency of 3 synthetic views coding, which are present in the PSNR-Bitrate graph in 1024 × 768 balloons and 1920 × 1088 Undo_Dancer sequences. The results also imply that the compression efficiency of Kendo and Balloons is better than that of Undo_Dancer and GT_Fly. These results prove that the coding efficiency depends on the specific sequences, and the numbers of distortionless base layer SDC-CUs are an important factor in influencing the performance of interlayer SDC.

## 5. Conclusion

We presented a scalable 3D video coding theme on the emerging HEVC, which supports two interlayer prediction methods on a multiloop decoder structure. The interlayer texture prediction method simultaneously exploits the interlayer correlation for texture views and depth maps. The interlayer SDC prediction tool achieves significant bitrate decrease and complexity reducing for the depth maps. Experimental results demonstrate the effectiveness of our proposed scenario. Improvement of the coding performance of scalable 3D video coding theme can be examined in future research. More interlayer prediction methods will be proposed to accomplish the scalability on 3D video coding.

## Figures and Tables

**Table 1:** Identifiers on interlayer SDC and traditional SDC.

Identifiers of the modes		Specified mode
Inter_SDC_Flag	SDC_Flag	
1	0	Interlayer SDC
0	1	Traditional SDC
0	0	Intracoded

**Table 2:** BD rate of inter-SDC prediction in multiloop to simulcast encoding.

Sequence	BD rate (%) of 2 synthesized views			BD rate (%) of 3 synthesized views		
	*Y*	*U*	*V*	*Y*	*U*	*V*
Kendo	55.7	63.6	56.5	53.5	70.6	55.6
Balloons	55.3	55.4	55.4	54.6	54.7	54.7
UDancer	63.3	60.2	59.9	58.5	59.3	58.1
GT_Fly	56.8	53.5	54.2	54.7	48.4	53.2
P_Street	44.9	47.9	38.8	50.9	51.3	47.8

Average	55.2	56.1	53.0	54.4	56.9	53.9
